# Importance of individual hemodynamic thresholds during exercise (*Hem-TRex*): relationship with blood pressure

**DOI:** 10.1038/s41371-026-01155-4

**Published:** 2026-05-06

**Authors:** Fabian Spahiu, Max Hagemann, Michelle Ottlik, Moritz Lampkemeyer, Christopher J. A. Pugh, Martin G. Schultz, Eric J. Stöhr

**Affiliations:** 1 COR-HELIX (Cardiovascular Regulation and Human Exercise Laboratory – Integration and Xploration), Institute of Sport Science, Leibniz University Hannover, Hannover, Germany; 2 Center for Cardiovascular Research Innovation and Development, Cardiff Metropolitan University, Cardiff, UK; 3 Menzies Institute for Medical Research, University of Tasmania, Hobart, TAS Australia; 4 Department of Rural Health, University of Melbourne, Shepparton, Australia

**Keywords:** Hypertension, Cardiovascular biology

Abnormal blood pressure (BP) responses to exercise are independently associated with cardiovascular disease. However, the physiological mechanisms underlying exercise BP are unclear, with traditional focus on examining differences in the slope (increase) of BP, or on the absolute maximal BP achieved during incremental exercise. Considering that cardiac function (stroke volume and myocardial deformation) and systemic hemodynamics (e.g, systemic vascular resistance, SVR) are known to reach their peak (i.e. plateau) at submaximal exercise [1, 2], different exercise BP progressions could be related to the individually varied attainment of peak hemodynamics. Indeed, young individuals with high BP (at rest) may be categorized into those that have a disproportionately high cardiac output (Q) and those who have a high systemic vascular resistance (SVR) [3]. Identifying the temporal attainment of peak Q and lowest SVR (i.e., their hemodynamic threshold during exercise, “Hem-TRex”) could, therefore, offer new insight to the physiology of exercise BP. Thus, the aim of this exploratory study was to determine individual Hem-TRex of Q and SVR, and their relationship with the Hem-TRex of BP. Based upon the previously suggested phenotypes, it was hypothesized that individuals who reach their peak Q early during incremental exercise would attain their lowest SVR later during incremental exercise, reflecting an inverse association, and that this may relate to exercise BP.

Participants (12 healthy young individuals;7 M, 5 F) completed two visits. Visit 1 determined W_peak_ during a maximal incremental test on a supine, tilted ergometer; Visit 2 consisted of 3-min stages starting at 40% of Visit-1 W_peak_ and increasing by 10% W_peak_ per stage until volitional exhaustion. Baseline brachial BP was assessed using an oscillometric cuff-based device (Mobil-O-Graph, IEM, Aachen, Germany) while BP during exercise was assessed using continuous finger photoplethysmography and corrected to generate brachial BP waveforms (NIBP, ADInstruments, Oxford, UK). Q was measured with 4-chamber echocardiography (GE, Vivid E95, Trondheim, Norway, and EchoPAC version 204, GE, Trondheim, Norway). SVR was calculated as MAP / Q · 80. VO_2_ was measured with breath-by-breath spirometry throughout the entire exercise test. Exercise data were collected during the last 90 seconds of each exercise stage. Key peak variables as well as data at Hem-TRex are shown in Table 1. Polynomial regression (quadratic or cubic models) was applied to characterize the hemodynamics in relation to the relative exercise effort (%HR_peak_). The optimal regression model for each parameter was selected based on the highest, corrected r² values and visual verification (quadratic models: *x* = −b/2a; cubic models: first derivative).

**Table 1:** Key variables at peak exercise and Hem-TRex.

	Peak	Hem-TRex (%HR_max_)
HR (bpm)	181 ± 12	100% (linear)
Workload (W)	226 ± 42	100% (linear)
VO_2_ (ml/min)	2887 ± 567	100% (linear)
RER	1.11 ± 0.05	100% (linear)
SBP (mmHg)	187 ± 26	85 ± 12%
DBP (mmHg)	80 ± 17	91 ± 13%
MAP (mmHg)	107 ± 22	88 ± 15%

**Fig. 1:**
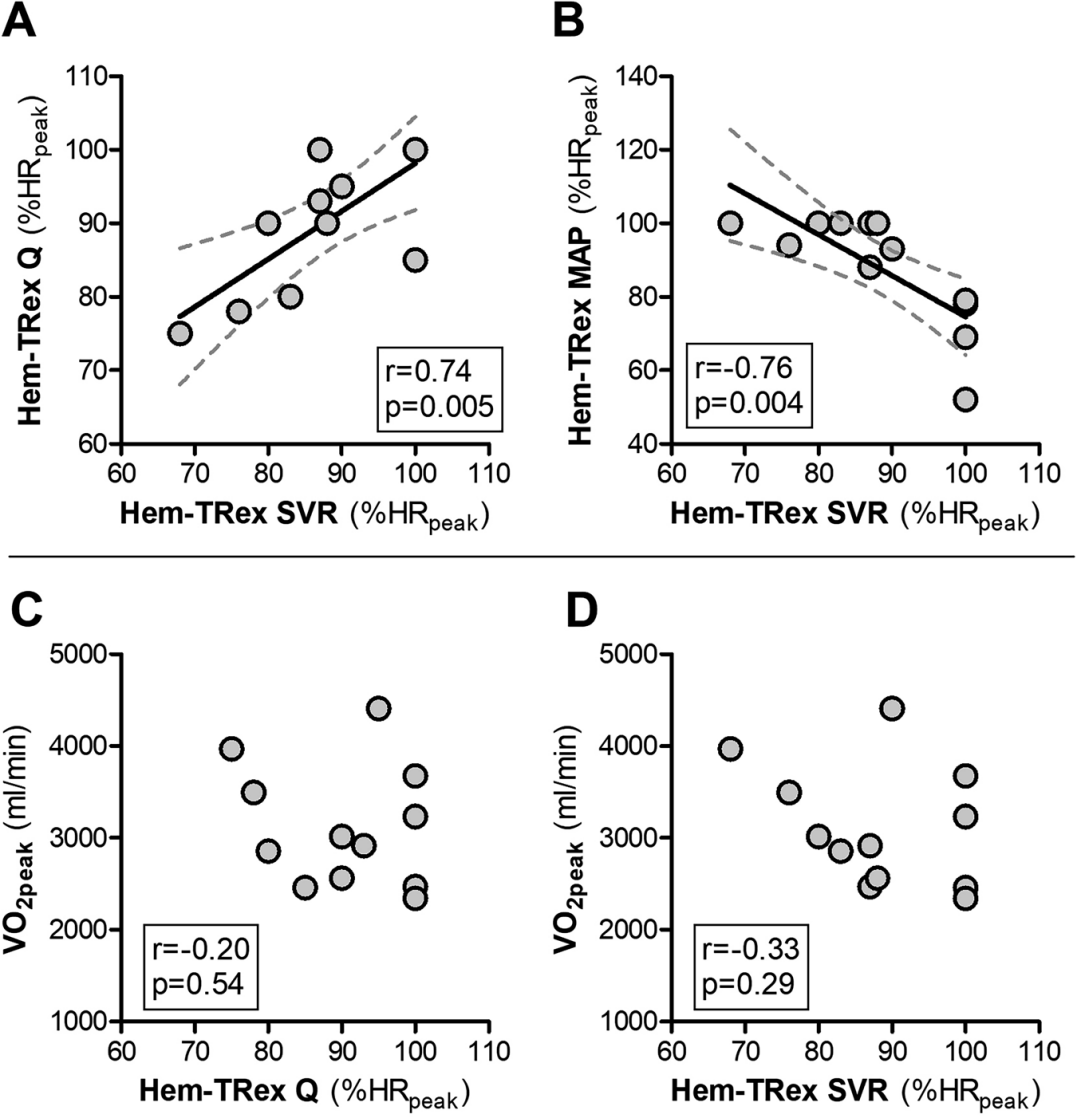
Temporal Sequence of Exercise Hemodynamics and Their Determinants. **A** An earlier attainment of the nadir of SVR during exercise was strongly associated with an earlier attainment of peak Q. **B** Conversely, earlier Hem-TRex SVR was negatively associated with Hem-TRex MAP. **C,D** Neither Hem-TRex Q nor Hem-TRex SVR correlated with VO_2_peak. The results suggest a finely-coordinated, interdependent time-course of hemodynamic progression during incremental exercise.

In this healthy cohort (age 26 ± 3 yrs; BMI 23.7 ± 2.5; brachial SBP 126 ± 12 mmHg; brachial DBP 81 ± 6 mmHg; MAP 100 ± 8 mmHg), 79% and 66% of individuals had a Hem-TRex SBP and Hem-TRex MAP, respectively, that occurred below maximal exercise effort. The temporal occurrences of Hem-TRex Q and SVR were positively associated (r = 0.74, p = 0.005, Fig. 1A), ranging from 75-100% and 68-100% for Hem-TRex Q and Hem-TRex SVR, respectively. Conversely, Hem-TRex MAP and Hem-TRex SVR were negatively associated (r = -0.76, p = 0.004, Fig. 1B), with MAP Hem-TRex ranging from 52-100% HR_peak_. Furthermore, there was no association between Hem-TRex Q and VO_2peak_ (Fig. 1B, C) or Hem-TRex SVR and VO_2peak_ (Fig. 1D). Moreover, the Hem-TRex of Q, SVR, and MAP did not correlate with the absolute Q, SVR, and MAP (r = 0.43, p = 0.16; r = 0.05, p = 0.87; r = 0.18, p = 0.58, respectively). Interestingly, the Hem-TRex of SBP did not correlate with the Hem-TRex of Q, SV, end-systolic volume nor the absolute SBP (r = 0.28, p = 0.38; r = 0.03, p = 0.91; r = 0.14, p = 0.70, r = 0.13, p = 0.68, respectively).

The results of this study strongly suggest an interdependent hemodynamic regulation that underpins exercise BP that has not been considered previously. Whilst some level of association between the time course of Q, MAP and SVR during exercise was expected, the results contrast the strict split into Q and SVR “phenotypes” previously proposed in individuals examined at rest only [3]. Instead, the positive association between Hem-TRex Q and Hem-TRex SVR indicates that – despite large differences in the time point of peak hemodynamics between individuals – there is a proportionate link between the temporal occurrence of Q and SVR during exercise. Since MAP Hem-TRex also correlated negatively with Hem-TRex SVR, it would appear that the temporal attainment of both cardiac output and arterial haemodynamics is inversely related to SVR. Uncovering the mechanisms of this unexplained phenomenon will potentially help to clarify the role of different haemodynamic “phenotypes” and the potential to modify these in accordance to lifestyle. Interestingly, in our study, aerobic fitness level did not seem to be a modifying factor. Nonetheless, it is possible that the observed relationships may be altered in the context of hypertension, when individuals may be less fit than those in the present study cohort [4]. Strikingly, the attainment of Hem-TRex MAP developed in the opposite time sequence to that of the nadir of SVR and peak Q. In other words, those individuals who reached their Hem-TRex Q early on during exercise also reached their lowest SVR earlier on, but with Hem-TRex MAP occurring proportionately later during exercise. Conversely, it seemed that individuals who had a Hem-TRex MAP at lower relative exercise intensities continued to rise their Q and SVR up to higher exercise effort. Since the temporal occurrence of these thresholds did not correlate with the absolute hemodynamics, it is possible that an altered time course of regulation – perhaps mediated by factors beyond sympathetic activity and baroreflex ‘resetting’ [5] – may underlie the changes in absolute hemodynamics in hypertensive individuals. Therefore, this study highlights that future examinations must consider the time course of hemodynamics during progressive exercise in addition to absolute hemodynamics to gain greater physiological understanding of exercise BP.
